# Supplementary material for: Organic vs. Non-Organic Plant-Based Foods—A Comparative Study on Phenolic Content and Antioxidant Capacity
Source: Plants (Basel). 2023 Jan 1;12(1):183. doi: 10.3390/plants12010183 (PMC9824154; doi:10.3390/plants12010183)
Supplement: Supplementary file 1 [file plants-12-00183-s001.zip › plants-2114330-supplementary.pdf]

Supplementary table S1. Agrometeorological parameters of organic and non-organic plant-based foods cultivation.

**Plant-based foods**		**Farming system**	**Agrometeorological parameters**						
			Average daily relative humidity (%)	Average daily temperature (°C)	Scalar wind speed at 2m (m/s)	Accumulated daily precipitation (mm/m²)	Daily global solar radiation (MJ/m²)	Reference evapotranspiration (mm/m²)	Altitude (m)
Fruits	Olive	NORG	70.0	19.9	1.4	241.0	21.8	4.0	132.0
		ORG	70.0	19.9	1.4	241.0	21.8	4.0	170.0
	Orange	NORG	71.8	21.2	1.3	478.6	20.5	3.8	134.0
		ORG	66.7	13.5	1.6	216.0	15.0	2.5	214.0
	Sweet cherry	NORG	63.7	12.4	0.0	129.0	n.d.	n.d.	263.0
		ORG	66.7	12.8	2.2	164.6	17.7	2.9	287.0
	Tomato E.	NORG	71.7	15.4	1.9	211.5	18.9	3.1	52.0
		ORG	70.5	22.7	1.3	164.2	23.3	4.4	366.0
	Tomato T.	NORG	71.7	15.4	1.9	211.5	18.9	3.1	52.0
		ORG	70.5	22.7	1.3	164.2	23.3	4.4	158.0
Vegetables	Onion	NORG	65.4	13.1	0.0	256.6	n.d.	n.d.	263.0
		ORG	70.9	22.8	1.3	159.0	23.9	4.5	374.0
	Sweet pepper	NORG	60.9	20.3	1.8	224.6	22.8	4.4	358.0
		ORG	70.6	22.6	1.3	297.0	23.0	4.3	24.0
	Swiss chard	NORG	68.5	12.7	1.6	342.4	9.9	1.5	158.0
		ORG	71.1	24.1	1.2	267.9	22.1	4.3	24.0
Nuts	Almond	NORG	73.1	19.8	1.6	686.0	19.8	3.7	112.0
		ORG	70.3	19.6	1.4	532.5	20.3	3.7	366.0
	Hazelnut C.	NORG	74.0	21.2	1.6	578.6	19.4	3.8	112.0
		ORG	71.7	20.9	1.6	300.8	22.4	4.2	104.0
	Hazelnut N.	NORG	71.3	22.4	1.3	430.7	21.1	4.0	366.0
		ORG	71.2	21.4	1.3	221.3	23.1	4.3	170.0
	Walnut	NORG	73.3	20.6	1.6	618.7	19.8	3.8	134.0
		ORG	71.1	21.6	1.3	450.5	21.3	4.0	366.0
Legume	Carob pod	NORG	73.3	20.6	1.6	618.7	19.8	3.8	112.0
		ORG	70.0	19.9	1.4	241.0	21.8	4.0	132.0

Abbreviations: Hazelnut C, hazelnut cv. Castanyera; Hazelnut N, hazelnut cv. Negreta; NORG, non-organic farming; ORG, organic farming; Tomato E, tomato cv. Ekstasis; Tomato T, tomato cv. Tores.
